# Relations between daily stressful events, exertion, heart rate variability, and thoracolumbar fascia deformability: a case report

**DOI:** 10.1186/s13256-024-04935-z

**Published:** 2024-12-02

**Authors:** Andreas Brandl, Raimund Engel, Christoph Egner, Robert Schleip, Christian Schubert

**Affiliations:** 1 Conservative and Rehabilitative Orthopedics, Department of Sport and Health Sciences, Technical University of Munich, Munich, Germany; 2 Vienna School of Osteopathy, Vienna, Austria; 3 Department for Medical Professions, Diploma Hochschule, Bad Sooden-Allendorf, Germany; 4 Department of Psychiatry, Psychotherapy, Psychosomatics and Medical Psychology, Medical University Innsbruck, Innsbruck, Austria

**Keywords:** Sport climbing, Integrative single case study, Case report, Time series analysis, Daily stressful events, Thoracolumbar fascia deformation

## Abstract

**Background:**

The posture of performance-oriented sport climbers adapts in a way that likely promotes the development of low back pain. The thoracolumbar fascia, while also contributing to performance, seems to be involved in that process. Furthermore, there has been evidence of the stiffening of the fascial structures in disorders associated with negative affectivity. The study aims to investigate the relationship between the autonomic nervous system and the deformability of the thoracolumbar fascia in a sport climber in a natural setting.

**Case presentation:**

Over a 30 day period, a 50-year-old German male reported daily morning resting heart rate variability and daily inventory of stressful events. The thoracolumbar fascia deformation was measured daily using ultrasound in a manual therapy clinic. A time series analysis was performed to detect possible time-delayed relationships between the variables.

A cluster analyses revealed two distinguishable heart rate variability clusters (heart rate variability clusters 1 and 2). Both clusters were correlated with thoracolumbar fascia deformation and daily inventory of stressful events. Higher heart rate variability cluster 1 immediately caused lower thoracolumbar fascia deformation. Heart rate variability cluster 1 parameters mediated the effect of daily inventory of stressful events on a decrease in thoracolumbar fascia deformation with a 2-day delay. One heart rate variability cluster 2 parameter mediated the effect of daily inventory of stressful events on an increase in thoracolumbar fascia deformation with a 7-day delay.

**Conclusion:**

Heart rate variability cluster 1 values, which probably indicate sympathetic nervous system activity, are directly related to thoracolumbar fascia. Presumably, the sympathetic nervous system mediated the effect of daily inventory of stressful events on a lower thoracolumbar fascia deformation with a 2-day delay, followed by a supercompensation, most likely parasympathetically mediated. Coaches and trainers should be aware of these mechanisms and consider monitoring during training to minimize potential additional risk factors for impaired performance and health.

**Supplementary Information:**

The online version contains supplementary material available at 10.1186/s13256-024-04935-z.

## Background

Sport climbing has established itself as an attractive recreational sport and a high-performance Olympic discipline in recent decades. It is assumed that sport climbing has a positive influence on low back pain (LBP) [1], but it could also promote its development due to postural adaptations in performance-oriented climbers over several years of intensive training [2–4].

Growing evidence suggests that the thoracolumbar fascia (TLF) has been a contributing factor to the development of LBP. In particular, biomechanical properties such as sliding or deformation are reduced in patients with LBP [5–8]. The number of nociceptive fibers in the aponeurotic TLF increases significantly due to inflammation [9] associated with hypersensitivity of these free nerve endings [10]. About 40% of the total TLF innervation consists of postganglionic sympathetic fibers, which are likely to be vasoconstrictors [11], potentially increasing pain levels under psychological stress as sympathetic activity is enhanced in such situations [12].

Heart rate variability is often used in sports to monitor and optimize training [13–15]. It is considered a biomarker that reflects the activity of the autonomic nervous system (ANS). A number of affective disorders are associated with changes in the ANS [12, 16, 17]. A recent study has revealed a possible role of myofascial tissue in the dynamics of depression maintenance. Individuals with major depressive disorder showed reduced elasticity and increased stiffness of this tissue compared with nondepressed controls. Interestingly, myofascial self-treatment of this stiffened tissue reduced negative memory bias and improved positive affect. Therefore, the authors concluded that the myofascial tissue potentially plays a role in dysfunctional mind–body dynamics [18].

By linking the findings on heart rate variability (HRV) as a measure of ANS activity, the strongly sympathetically innervated TLF and the role of myofascial tissue in affective disorders, the question arises as to whether there is a connection between these mechanisms, which is particularly interesting in sport climbing. The TLF could act here as a supporting structure for the back muscles and contribute to their performance [19, 20].

This relationship is thought to be highly dynamic and the HRV and/or tissue response to a stressor may not be immediate but delayed [14]. Therefore, an *n*-of-one study focusing on a single athlete in a specific time period provides better insights into the intricate dynamics of complex biopsychosocial responses than a randomized controlled trial with a selective focus on the characteristics of a specific sample [21]. In general, an integrative single case design was used to investigate the dynamics of the ANS and the connective tissue system under training conditions. This approach, in which biopsychosocial time series of an individual are analyzed, has already revealed bidirectional interdependencies between fatigue, mood, and activity of the cellular immune system, as well as oxidative stress in several studies [22–25].

The aim of this case report is to investigate this relationship during a typical mesocycle in the climber's “life as it is lived.” It was hypothesized that a shift in ANS reflected in HRV would result in a measurable TLF response. Furthermore, that daily stressful events, mood, or exertion would lead to changes in ANS activity, which mediates its effect on the TLF indirectly.

## Case presentation

This case is an integrative single-case study from the Associations of Heart Rate Variability and Tissue Characteristics (AHAT) project, in which the relationships between HRV and tissue characteristics is investigated using a time-series *n*-of-one approach (German Register of Clinical Trials: DRKS00033489). It complied with CARE guidelines and the Declaration of Helsinki [26] and was approved by the local ethics committee. The participant provided written informed consent.

The participant was a 50 year-old German (male, height 1.85 m, weight 74 kg, body mass index 21.62 kg/m^2^, body fat: 11.5%) moderate performance-oriented sport climber [2]. The Union Internationale des Associations d’Alpinisme (UIAA) climbing level was 9 [27]. The observation period lasted 30 days, which corresponded to a mesocycle with a weekly workload of 9.0 ± 1.2 hours. The participant had 25 years of climbing experience.

### Ultrasound measurement of the deformation of the thoracolumbar fascia

The TLF deformation (TLFD) was measured similar to Brandl *et al*. [20], described previously. The protocol is in detail documented and available at https://doi.org/0.17504/protocols.io.eq2lyjbmwlx9/v1. Briefly, this involves the participant sitting on a treatment table and flexing the trunk to an angle of 60°, which is the starting position for taking an ultrasound image. Subsequently, he extends the trunk to an angle of 0°, the ending position in which the second ultrasound image is taken. Measurement is made of the distance between the latissimus dorsi muscle/TLF transition and an artificial shadow created by a reflective tape on the skin shown in the images. Therefore, this difference between the distance of the starting position and the ending position is the extent of the TLFD. The method has shown excellent intrarater reliability (intraclass correlation coefficient; ICC of 0.92) and moderate interrater reliability (ICC of 0.78) [28].

### Heart rate variability

The HRV was assessed using a chest strap device (Polar H10; Polar Electro Oy, Kempele, Finland, sampling rate 1000 Hz; app software Elite HRV App, Version 5.5.1, Asheville, NC) according to the standardization checklist of procedures [29]. The Polar H10 sensor was proven to be valid with an ICC value of 0.95 in the resting state [13]. The evaluation included time-domain, frequency-domain, and nonlinear parameters. Table 1 presents the assumed ANS characteristics of the individual HRV parameters and suggested interpretation according to [30] and Gullett *et al*. [17]. However, this is probably an oversimplification that does not reflect the complex self-referential interrelationship between sympathetic nervous system (SNS) and parasympathetic nervous system (PNS) activity. The HRV data were recorded for 5 minutes immediately after waking up in the morning. Data analysis was performed using Kubios HRV software (version 3.5.0). This method of HRV assessment has been reported to have excellent reproducibility for short-term measurements (ICC of 0.95–0.99; Plaza-Florido *et al*. [31]).

**Table 1:** Heart rate variability parameters

Parameter	Unit	Description and assumed ANS characteristics
Time domain parameters		
SDNN	Milliseconds	Standard deviation of the RR intervals; sympathetic and parasympathetic tone
RMSDD	Milliseconds	Root mean square of successive differences; predominant parasympathetic tone
pNN50	%	Number of successive RR interval pairs that differ more than 50 ms divided by total number of RR intervals; predominant parasympathetic tone
Frequency domain parameters		
LF	Square milliseconds	Power of the low-frequency band; sympathetic and parasympathetic tone
HF	Square milliseconds	Power of the high-frequency band; parasympathetic tone
Non-linear parameters		
SD1	Milliseconds	Poincaré plot standard deviation perpendicular to the line-of-identity; parasympathetic tone
SD2	Milliseconds	Poincaré plot standard deviation along the line-of-identity; long term HRV; sympathetic and parasympathetic tone

### Self-reported outcomes

Daily stressors were evaluated using the daily inventory of stressful events (DISE; Almeida *et al*. [32]. The participant responded to inquiries concerning disagreements, potential conflicts, stressors at work/volunteer engagements and home, network stressors (pertaining to friends and family), health-related incidents, and other stressors each day. The internal consistency of the test was reported with a Cronbach’s *α* of 0.88 and a reliability coefficient of 0.82 [33].

The category ratio scale (CR-10), is a subjective rating scale commonly used in exercise physiology and sports science to assess an individual’s perceived level of exertion or effort during physical activity. The scale was developed by Gunnar Borg [34], a Swedish psychologist, as an extension of his earlier rating of perceived exertion (RPE) scale. The CR-10 consists of a series of numerical values ranging from 0 to 10, with corresponding verbal anchors that describe the perceived exertion at each point on the scale. The scale is designed to be simple and easy to use. Participants are asked to choose a number from the scale that best represents their perceived exertion, where 0 indicates no exertion at all and 10 represents maximal exertion or the highest level of effort imaginable. Reliability for the scale was reported high (*r* = 0.90).

To assess mood, the state-trait anxiety inventory six-item short form (STAI-6; Cronbach’s *α* = 0.82) was used. This scale contains 6 items that are rated on a Likert scale of 1–4. Scores between 20 and 80 can be achieved [35].

### Procedure

The mesocycle with a focus on maximum performance and speed lasted 4 weeks. The weekly training included two bouldering sessions of 2.5 hours each and two lead sessions of 2 hours each. Prior to the cycle, the participant completed the Physical Activity Readiness Questionnaire (PAR-Q; Thomas *et al*. [36] and underwent a sports medical examination with resting and exercise electrocardiography. He was already using the Polar H10 HRV sensor with the Elite HRV app before the study period and was familiar with the procedure. HRV data were collected every day immediately after waking up at approximately 7:00 am (± 15 minutes). Room temperature and humidity were controlled and ranged from 15 °C to 18 °C and 42% to 49% humidity respectively. He then documented the self-reported outcomes. The TLFD was measured at 7:30 in a manual therapy clinic within walking distance.

### Statistical analysis

First, a Bonferroni-adjusted hierarchically clustered Spearman correlation matrix from the R package corrplot with default settings was used to determine HRV parameter groups [37].

Second, a cross-correlation function (CCF) was performed using the R package tseries to determine possible time lags of up to 7 days between HRV and TLFD as well as the self-reported outcomes and HRV [22–25]. For this purpose, the stationarity of the time series was tested with the Kwiatkowski–Phillips–Schmidt–Shin test, prewhitened by centered moving average (CMA) smoothing when autocorrelations were significant according to the Durbin Watson test [38], and the largest correlation coefficients that reached the significance of *p* < 0.05 were defined as criteria for determining the time lag.

Third, a multiple lagged linear regression model (LM) was applied for each significant CCF to test the relationship between HRV and TLFD, as well as between self-reported outcomes and HRV, controlling for respiratory frequency. Standardized 95% confidence intervals (CIs) and *p* values (Bonferroni adjusted for each HRV cluster and lag) were calculated for the analysis. According to Cohen (1988), the resulting *R*^2^ values were interpreted as “weak” (0.02–0.13), “moderate” (0.13–0.26), or “substantial” (0.26–1.0).

Fourth, significant lagged TLFD and HRV predictors in the LM were tested with the Granger causality test to determine whether the predictor or the response variable causes the other [38, 40].

Fifth, the potential HRV-mediated influence of self-reported outcomes (which were significant predictors of HRV in the LM) on TLFD was tested by mediation analysis using the R package lavaan. Therefore, significant TLFD predicting HRV variables in the LM were used as mediators for self-reported outcomes to predict TLFD (self-reported outcomes → HRV → TLFD).

All analyses were carried out using the software R, version 4.3.2 (R Foundation for Statistical Computing, Vienna, Austria). Time series plots were generated in Matlab, version R2023b (The MathWorks Inc., Natick, MA).

## Results

**Fig. 1:**
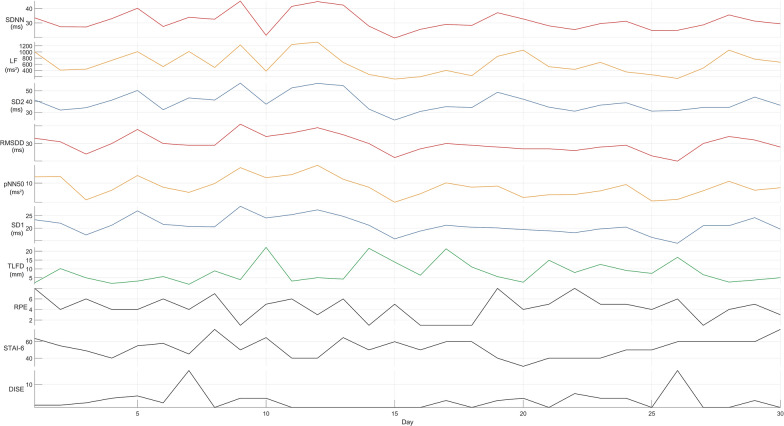
Time series of all measured variables. *SDNN* standard deviation between RR intervals, *LF* low frequency band power, *SD2* Poincaré parallel standard deviation, *RMSSD* root mean square of successive differences, *pNN50* percentage of successive RR intervals that deviate greater than 50 ms; SD1, Poincaré perpendicular standard deviation, *TLFD* deformation of thoracolumbar fascia, *RPE* rating of perceived exertion, *STAI-6*, State-Trait Anxiety Inventory six-item short form, *DISE* daily inventory of stressful events

A total of 30 measurements were taken on consecutive days (*n* = 30) and no data were missing. There was no sign of disease or infection, and the participant was not using medication, drugs, or alcohol. The descriptive statistics for TLFD, RPE, STAI-6, and DISE are presented in Table 2. Figure 1 shows the raw data of the time series of all measured variables.

**Table 2:** Descriptive statistics

Percentiles						
	*N*	Mean	SD	25th	50th (median)	75th
TLFD	30	8.2	6.07	3.79	6.08	10.86
RPE	30	4.37	2.16	3.25	4.5	6
STAI-6	30	52.87	11.13	41.25	52.5	60
DISE	30	2.73	4.08	0	1.5	4

*TLFD* deformation of thoracolumbar fascia, *RPE* rating of perceived exertion, *STAI-6* State-Trait Anxiety Inventory six-item short form, *DISE* daily inventory of stressful events

**Fig. 2:**
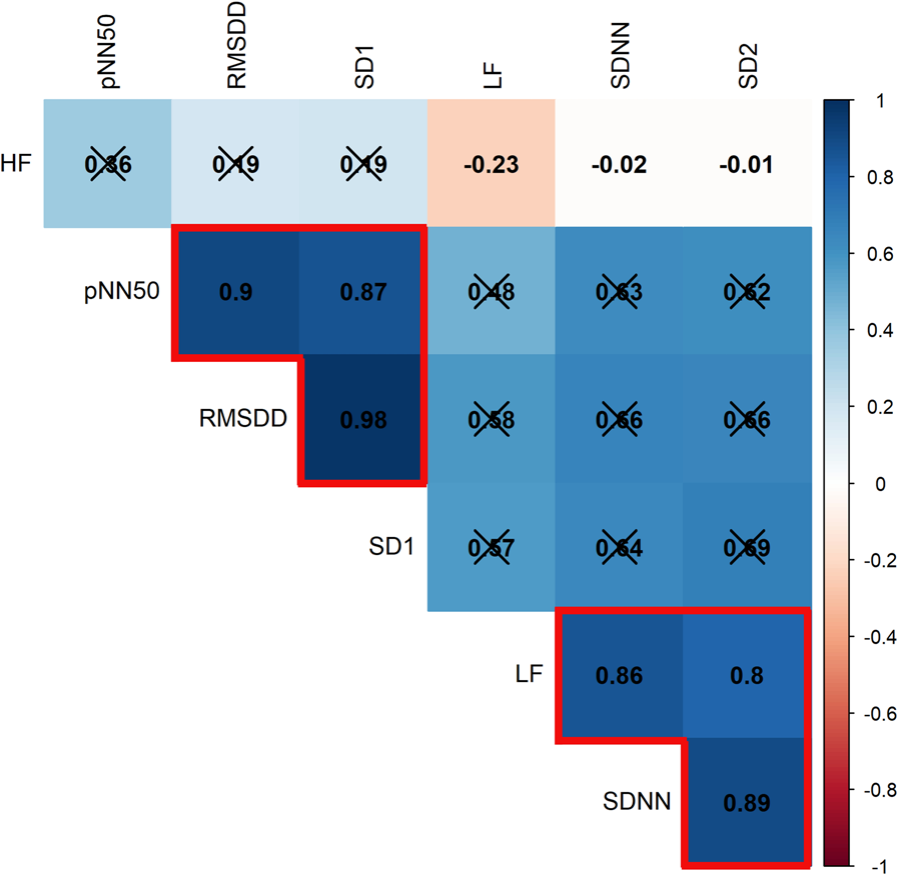
Hierarchically clustered correlation matrix of heart rate variability parameters. The color and size of the squares indicate the correlation strength, with blue shading indicating positive correlations and red shading indicating negative correlations. The numbers in the squares represent the Spearman correlation coefficient. Crossed-out squares indicate non-significant correlations (p_adj._ < .00714). The squares outlined in red show the grouping based on the correlations between the individual variables, which are grouped into heart rate variability cluster 1 (HRV-1; bottom right) and heart rate variability cluster 2 (HRV-2; top left). *SD1* Poincaré perpendicular standard deviation, *SD2* Poincaré parallel standard deviation, *SDNN* standard deviation between RR intervals, *LF* low frequency band power, *HF* high frequency band power, *pNN50* percentage of successive RR intervals that deviate greater than 50 milliseconds, *RMSSD* root mean square of successive differences

The Bonferroni-adjusted hierarchical clustered Spearman correlation matrix showed two different clustered parameter sets across the time frequency and the nonlinear HRV measures (HRV1: SDNN, SD2, LF and HRV2: RMSDD, pNN50, SD1; Fig. 2).

Since previous studies suggested that the parameters in the HRV1 cluster, although not exclusive, reflect to a certain extent the tone of the SNS and in the HRV2 cluster the tone of the PNS, it was assumed that a shift between these cluster parameters is also an indication of a shift in the autonomic nervous system [17, 30].

### HRV1 and TLFD parameters

**Fig. 3:**
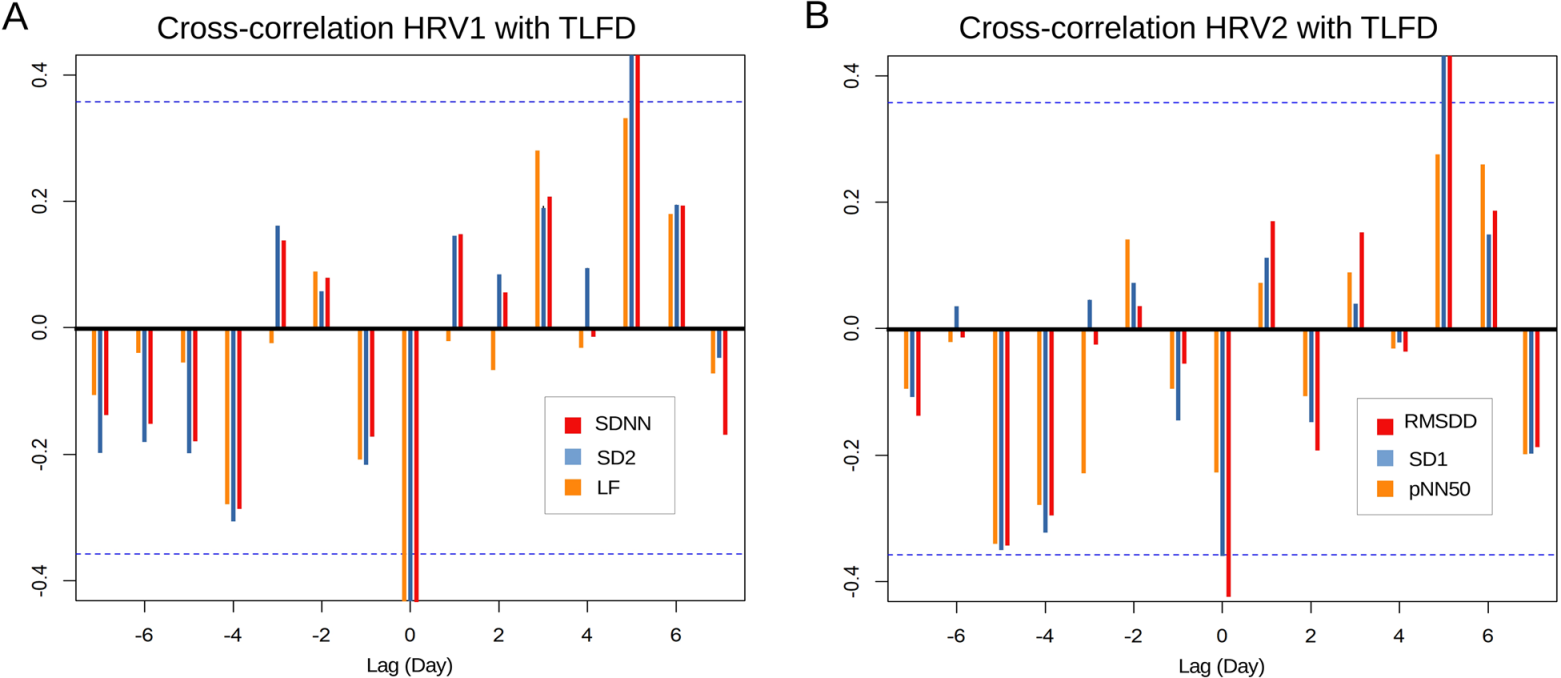
Time series analysis of heart rate variability parameters. **A** Cross-correlation of heart rate variability cluster 1 with thoracolumbar fascia deformation. Heart rate variability cluster 1 showed an immediate negative correlation (lag 0) with standard deviation between RR intervals, Poincaré parallel standard deviation, and low frequency band power. Furthermore, the first heart rate variability cluster correlated positively 5 days later (lag 5). **B** Cross-correlation of the second heart rate variability cluster with thoracolumbar fascia deformation. The second heart rate variability cluster showed an immediate negative correlation (lag 0) with RMSDD and was correlated positively 5 days later (lag 5) with RMSDD and SD1. *HRV1* first heart rate variability cluster, *HRV2* second heart rate variability cluster, *TLFD* deformation of thoracolumbar fascia, *SDNN* standard deviation between RR intervals, *SD2* Poincaré parallel standard deviation, *LF* low frequency band power, *RMSSD* root mean square of successive differences, *pNN50* percentage of successive RR intervals that deviate greater than 50 milliseconds, *SD1* Poincaré perpendicular standard deviation

Figure 3A shows the CCF between HRV1 and TLFD. All HRV1 parameters showed a significant negative correlation at lag 0 with TLFD (SDNN: *r* = −0.577, *p* = 0.002; SD2: *r* = −0.506, *p* = 0.006; LF: *r* = −0.650, *p* < 0.001). Both, SDNN and SD2 showed a positive correlation at lag 5 with TLFD (SDNN: *r* = 0.438, *p* = 0.016; SD2: *r* = 0.500, *p* = 0.006).

The results of the LM with HRV1 at lag 0 and lag 5 as predictors of the squared transformed TLFD (to meet the criteria for parametric testing) revealed significant effects. There were significant correlations between higher SDNN and SD2, as well as LF values and lower TLFD at time lag 0 (all *p* < 0.002) and between higher SD2 and higher TLFD at lag 5 (*p* = 0.02). Table 3 presents the results of the linear regression analyses for HRV1 (additional figures for the analysis can be found in the Supplementary Material). The controlled covariate respiratory frequency had no significant effect in any model (TLFD ~ SDNN(lag 0): *B *= 0.01, 95% CI [−0.14 to 0.16], *p* = 0.896; TLFD ~ SD2(lag 0): *B* = 0.03, 95% CI [−0.14 to 0.19], *p* = 0.751; TLFD ~ LF(lag 0): *B* = 0.034, 95% CI [−0.09 to 1.16], *p* = 0.589; TLFD ~ SDNN(lag 5): *B* = 0.02, 95% CI [−0.16 to 0.21], *p* = 0.800; TLFD ~ SD2(lag 5): *B* = 0.04, 95% CI [−0.14 to 0.22], *p* = 0.656).

**Table 3:** Linear regression modeling for HRV1 predicting TLFD^1^

						95% CI			
Predictor	Lag	*F*	*R*^2^	Formula	SE	Lower	Upper	*t*	p^*^
SDNN	0	7.56	0.36	5.43–0.09*x*	1.45	−0.15	−0.04	−3.46	**0.006**
	5	2.96	0.21	0.33 + 0.07*x*	0.03	0.01	0.14	2.25	0.07
SD2	0	4.76	0.26	4.61–0.06*x*	0.02	−0.1	−0.01	−2.59	**0.045**
	5	4.32	0.28	−0.14 + 0.06*x*	0.02	0.02	2.77	2.77	**0.03**
LF	0	13.7	0.5	3.52–0.01*x*	0.01	−0.01	−0.01	−4.81	** < 0.001**

*HRV1* HRV cluster 1, *TLFD* deformation of thoracolumbar fascia, *95% CI* 95% confidence interval, *F* overall model test, *SE* standard error, *t* t-statistic, *SDNN* standard deviation between RR intervals; *SD2* Poincaré parallel standard deviation, *LF* low frequency band power. ^*^ Bonferroni adjusted *p* value

^a^TLFD was squared transformed to meet the criteria for parametric testing. Significant results are printed in bold

The participant’s TLFD was not significantly Granger-caused by SD2 at lag 5 (*F* = 1.35, *p* = 0.301). Bivariate analysis revealed also no Granger causality in the other direction, meaning that SD2 was not caused by TLFD (*F* = 1.93, *p* = 0.153).

### HRV2 and TLFD parameters

Figure 3B shows the CCF between HRV2 and TLFD. RMSDD showed a significant negative correlation at lag 0 with TLFD (*r* = −0.423, *p* = 0.021). Both RMSDD and SD1 showed a positive correlation at lag 5 with TLFD (RMSDD: *r* = 0.487, *p* = 0.008; SD1: *r* = 0.517, *p* = 0.005).

The results of the LM between HRV2 at lag 0 as predictor of the squared transformed TLFD (to meet the criteria for parametric testing) revealed no significant effects (all *p* > 0.05). At lag 5, RMSDD and SD1 correlated positively with TLFD. There were significant correlations between higher RMSDD and SD1 values and higher TLFD at lag 5 (all *p* < 0.002). Table 4 presents the results of the linear regression analyses for HRV2 (additional figures for the analysis can be found in the Supplementary Material). The controlled covariate respiratory frequency had no significant effect in any model (TLFD ~ RMSDD(lag 5): B = −0.01, 95% CI [−0.17 to 0.16], *p* = 0.962; TLFD ~ SD1(lag 5): *B* = −0.01, 95% CI [−0.17 to 0.16], *p* = 0.967).

**Table 4:** Linear regression modeling for HRV2 predicting TLFD^1^

						95% CI			
Predictor	Lag	*F*	*R*^2^	Formula	SE	Lower	Upper	*t*	p^*^
RMSDD	0	2.56	0.16	3.45–0.06*x*	0.08	−0.14	0.02	−1.62	0 .120
	5	4.65	0.3	−0.58 + 0.11*x*	0.04	0.03	0.2	2.89	**0.017**
SD1	5	4.36	0.28	−0.56 + 0.16*x*	0.06	0.04	0.28	2.79	**0.022**

*HRV2* HRV cluster 2, *TLFD* deformation of thoracolumbar fascia, *95% CI* 95% confidence interval, *F* overall model test, *SE* standard error, *t* t-statistic, *RMSSD* Root mean square of successive Differences; *SD1* Poincaré perpendicular standard deviation.

^*^Bonferroni adjusted *p* value

^a^TLFD was squared transformed to meet the criteria for parametric testing. Significant results are printed in bold

The participant’s TLFD was significantly Granger-caused by RMSDD at lag 5 (*F* = 3.22, *p* = 0.038). Bivariate analysis revealed no Granger causality in the other direction, meaning that RMSDD was not caused by TLFD (*F* = 0.85, *p* = 0.539). TLFD was significantly Granger-caused by SD1 at lag 5 (*F* = 3.96, *p* = 0.012), and SD1 was not caused by TLFD (*F* = 0.94, *p* = 0.486).

### Self-reported outcomes and HRV1 parameters

There were significant correlations at lag 5 of RPE with HRV1(all *p* < 0.023). RPE showed a significant negative correlation with all HRV1 parameters (SDNN: *r* = −0.437, *p* = 0.017; SD2: *r* = −0.468, *p* = 0.010; LF: *r* = −0.417, *p* < 0.023); an additional figure for the analysis can be found in the Supplementary Material). The results of the LM with RPE at lag 5 as predictor for HRV1 revealed no significant effects (all *p* > 0.05; an additional table for the analysis can be found in the supplementary material).

There were no significant correlations at any time lag between STAI-6, and HRV1 (all *p* > 0.05; an additional figure for the analysis can be found in the Supplementary Material).

**Fig. 4:**
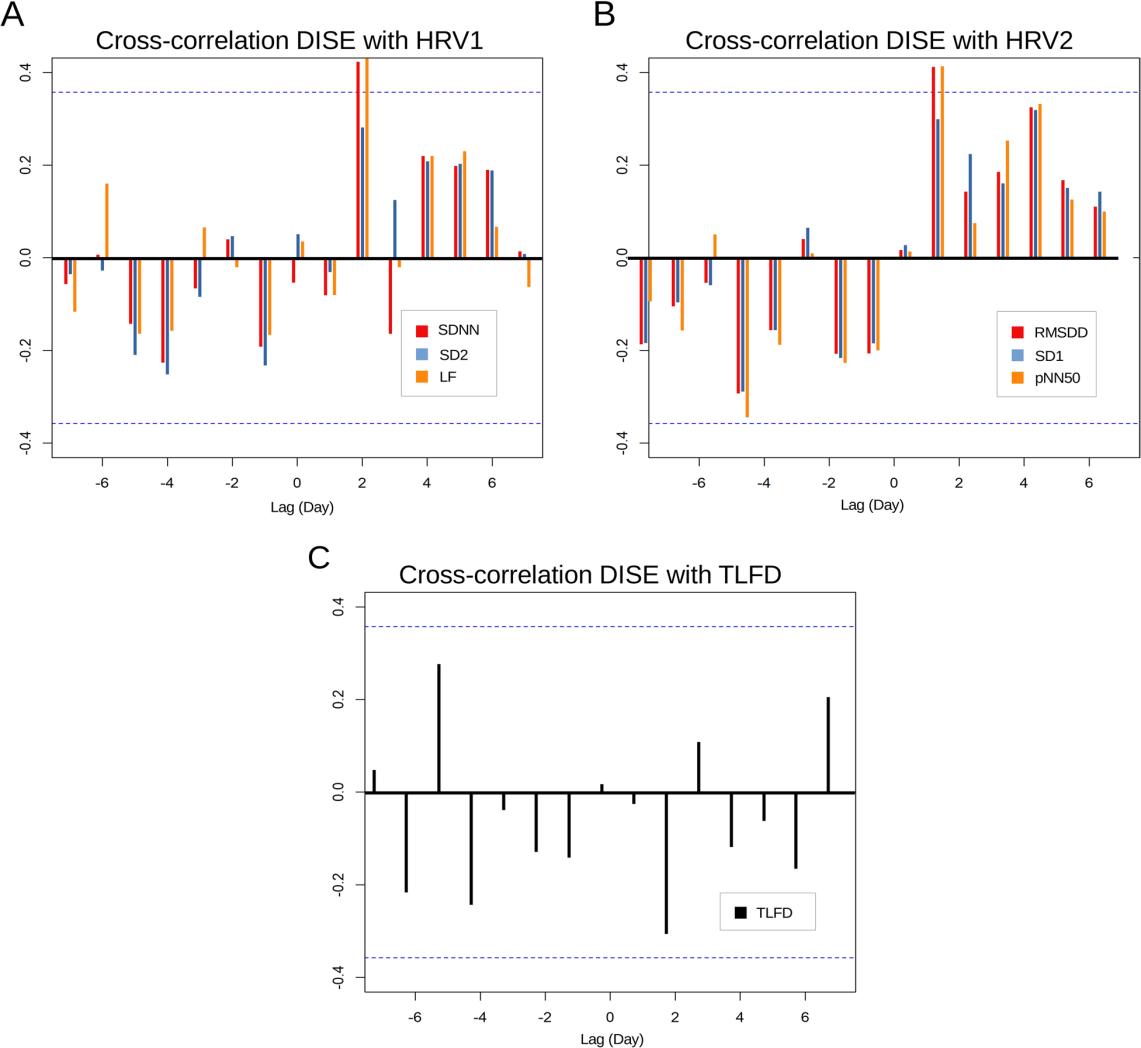
Cross-correlation of daily inventory of stressful events with heart rate variable and deformation of thoracolumbar fascia. **A** Cross-correlation of daily inventory of stressful events with heart rate variable cluster 1. Daily inventory of stressful events showed a positive correlation 2 days later (lag 2) with SDNN and LF. **B** Cross-correlation of daily inventory of stressful events with heart rate variable cluster 2. Daily inventory of stressful events showed a positive correlation 2 days later (lag 2) with RMSDD and pNN50. **C** Cross-correlation of daily inventory of stressful events with deformation of thoracolumbar fascia. Daily inventory of stressful events showed a nonsignificant trend (*p* = 0.072) toward a negative correlation 2 days later (lag 2) with deformation of thoracolumbar fascia. *DISE* daily inventory of stressful events, *HRV1* first heart rate variable cluster, *HRV2* second heart rate variable cluster, *TLFD* deformation of thoracolumbar fascia, *SDNN* standard deviation between RR intervals, *SD2* Poincaré parallel standard deviation, *LF* low frequency band power, *RMSSD* root mean square of successive differences, *pNN50* percentage of successive RR intervals that deviate greater than 50 milliseconds, *SD1* Poincaré perpendicular standard deviation

DISE showed a significant positive correlation with SDNN at lag 2 (*r* = 0.423 *p* = 0.021) and LF (*r* = 0.443, *p* = 0.016) in the CCF. SD2 (all *p* > 0.13) was not significant at any time lag (Fig. 4A). With a rating of 16, two events exceeded the other days almost threefold. The participant reported an argument with his daughter on day 7 and one with his wife on day 26 of the study. Both events were described by the participant as very meaningful and with considerable negative psychological effects.

The results of the LM with DISE as predictor of HRV1 at lag 2 revealed significant effects. There were significant correlations between higher DISE and higher SDNN as well as LF values 2 days later. Table 5 presents the results of the LM for DISE and HRV1 (an additional figure for the analysis can be found in the Supplementary Material). The controlled covariate respiratory frequency had a significant effect in the SDNN ~ DISE LM (*B* = −1.38, 95% CI [−2.34 to −0.42], *p* = 0.014) but not in the LF ~ TLFD LM (*B* = −57.2, 95% CI [−11.08 to −0.41], *p* = 0.076).

**Table 5:** Linear regression modeling for DISE predicting HRV1 at lag 2

					95% CI			
DV	F(2,25)	*R*^2^	Formula	SE	Lower	Upper	*t*	p^*^
SDNN	8.15	0.4	45.45 + 0.61*x*	0.25	0.09	1.12	2.43	**0 .044**
LF	6.35	0.34	1178.5 + 35.8*x*	14	7.05	64.56	2.56	**0 .034**

*HRV2* HRV cluster 2, *DISE* daily inventory of stressful events, *95% CI* 95% confidence interval, *DV* dependent variable, *F* overall model test, *SE* standard error, *t* *t*-statistic, *SDNN* standard deviation between RR intervals, *LF* low frequency band power

^*^Bonferroni adjusted *p* value. Significant results are printed in bold

### Self-reported outcomes and HRV2 parameters

There were no significant correlations at any time lag between RPE, STAI-6, and HRV2 (all *p* > 0.05; additional figures for the analysis can be found in the Supplementary Material)).

DISE showed a significant positive correlation with the HRV2 parameter RMSDD at lag 2 (*r* = 0.411, *p* = 0.024) and pNN50 (*r* = 0.410, *p* = 0.025). SD1 (*p* > 0.10) was not significant correlated with DISE (Fig. 4B).

The results of the LM with DISE as a predictor of HRV2 lag 2 revealed no significant effects (all *p* > 0.06). Table 5 presents the results of the LM for DISE and HRV2 (an additional figure for the analysis can be found in the Supplementary Material). The controlled covariate respiratory frequency had no significant effect in any model (RMSDD ~ DISE: *B* = −0.53, 95% CI [−1.35 to 0.30], *p* = 0.201; pNN50 ~ DISE: *B* = −0.53, 95% CI [−1.35 to 0.30], *p* = 0.202).

### HRV-mediated influence of self-reported outcomes on TLFD

Simple mediation analyses were performed using the HRV1 parameters SDNN, SD2, and LF as mediator for DISE as predictor of the squared transformed TLFD (to meet the criteria for parametric testing) 2 days later (lag 2) and SD2 as mediator for DISE as predictor of TLFD 7 days later (lag 7). There was no significant direct effect of DISE on TLFD (all *p *> 0.212). SDNN, SD2, and LF mediated significantly the effect of DISE on TLFD (Table 6). An additionally conducted CCF showed a trend toward a negative correlation between DISE and TLFD at lag 2 (*r* = 0.303; *p* = 0.072; Fig. 4C).

**Table 6:** Mediation analysis for HRV1 as mediator between DISE and TLFD

					95% CI			
Parameter	Lag	*B*	SE	Lower	Upper	*Z*	*p*	Percent mediation
SDNN	2	-0.06	0.29	−0.12	−0.01	−2.02	**0.044**	75.2
SD2	2	−0.03	0.02	−0.07	0.02	−1.26	**0.021**	36.2
	7	0.38	0.22	−0.06	0.82	1.69	0.09	67.9
LF	2	−0.08	0.03	−0.14	−0.01	−2.32	**0.020**	97.58

*HRV1* HRV cluster 1, *DISE* daily inventory of stressful events, *TLFD* deformation of thoracolumbar fascia, *95% CI* 95% confidence interval, B estimate, *SE* standard error, *Z* *Z*-statistic, *SDNN* standard deviation between RR intervals, *SD2* Poincaré parallel standard deviation, *LF* low frequency band power. Significant results are printed in bold

Simple mediation analyses were performed using the HRV2 parameters RMSDD and SD1 as mediator for DISE as predictor of TLFD 7 days later (lag 7). There was no significant direct effect of DISE on TLFD (all *p* > 0.155). RMSDD and SD1 mediated significantly the effect of DISE on TLFD (Table 7).

**Table 7:** Mediation analysis for HRV2 as mediator between DISE and TLFD

					95% CI			
Parameter	Lag	*B*	SE	Lower	Upper	*Z*	*p*	Percent mediation
RMSDD	7	0.5	0.25	0.01	1	1.98	**0.048**	90.26
SD1	7	0.48	0.25	−0.01	0.97	1.94	0.052	87.1

*HRV2* HRV cluster 2, *DISE* daily inventory of stressful events, *TLFD* deformation of thoracolumbar fascia, *95% CI* 95% confidence interval, *B* estimate, *SE* standard error, *Z* *Z*-statistic, *RMSSD* root mean square of successive differences, *SD1* Poincaré perpendicular standard deviation. Significant results are printed in bold

**Fig. 5:**
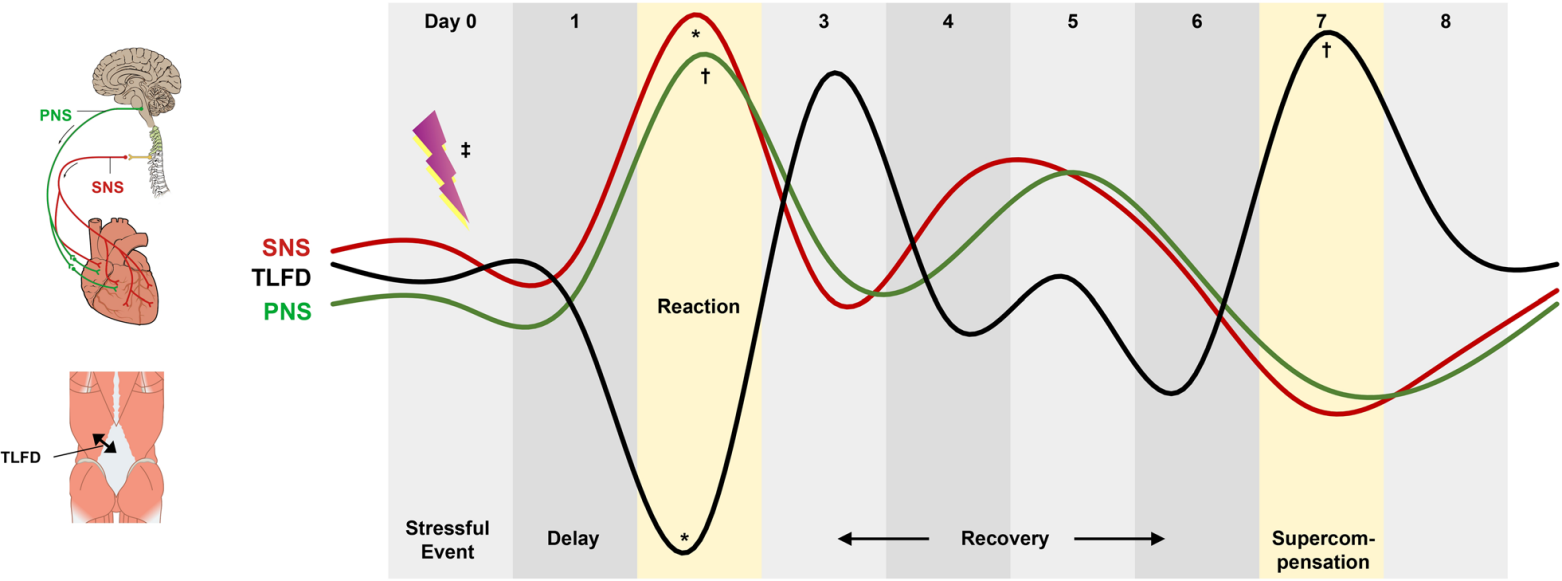
Schematic representation of the observed 24 hour changes in autonomic nervous system and deformation of thoracolumbar fascia. The diagram is based on the centered moved average-smoothed residuals of the time series. Day 0 marks the daily stressful event corresponding to lag 0, day 2 marks the ANS/deformation of thoracolumbar fascia response at lag 2 and day 7 marks the deformation of thoracolumbar fascia supercompensation at lag 7. *ANS* autonomic nervous system, *TLFD* deformation of thoracolumbar fascia, *SNS* sympathetic nervous system, *PNS* parasympathetic nervous system, *CMA* centered moved average. ^*^ Significant association between higher sympathetic nervous system and lower deformation of thoracolumbar fascia with a lag of 0; ^†^ Significant association between higher PNS and higher deformation of thoracolumbar fascia with a lag of 5. ^‡^ Sympathetic nervous system significantly mediated the effect of the daily stressful event 2 days later negatively on deformation of thoracolumbar fascia (Reaction), PNS significantly mediated the effect of the daily stressful event 7 days later positively on deformation of thoracolumbar fascia (supercompensation). The figure has been modified from OpenStax College (2013). Anatomy and physiology. http://cnx.org/content/col11496/latest. CC BY 4.0 license

Figure 5 shows an illustrated summary of the observed HRV and TLFD outcomes.

## Discussion

The work presents an integrative single-case study of a 50-year-old, moderate performance-oriented sport climber in a typical mesocycle of training. To the authors’ knowledge, this was the first study to investigate the complex relationships between ANS and tissue characteristics using a combination of CCF, multivariate LM taking into account time-delayed effects and cause-effect relationships between 30 and 24 hour measurements.

A cluster analysis of the derived HRV parameters revealed two clearly distinguishable clusters. Following the studies of several authors, we related HRV1 to SNS activity and HRV2 to PNS activity [14, 41–43] examined HRV at rest after stressful events in 30 military athletes and performed the same cluster analysis. The analysis revealed identical clusters as in the present study; therefore, it is assumed that both clusters represent different aspects of the ANS.

All HRV1 parameters were significantly negatively correlated with TLFD at the same time. LF showed the best goodness-of-fit index and could explain 50% of the variance in the LM. In literature, LF is described as one of the HRV parameters that probably best reflects SNS activity [14, 41, 42]. However, it is also influenced by the PNS (see “Limitations” section for a detailed discussion). During the study period, a TLFD drop of up to 19 millimeters was observed from 1 day to the next, accompanied by a threefold increase in LF. Interestingly, although the RPE showed no significant effect on TLFD in the LM, the largest drop was observed on 2 days of intense bouldering sessions, which were categorized as “very hard” by the participant. A study by Maspers *et al*. [43] investigated the influence of the SNS on fluid filtration during intense exercise in cats. They found that an exercise-induced increase in capillary pressure, which led to filtration of plasma fluid into the interstitial tissue, was counteracted by increased SNS activity. It has also been described that increased SNS activity enhances vascular permeability in subcutaneous adipose tissue [44]. It is likely that these mechanisms significantly alter the fluid dynamics between the layers of TLF and their vicinity. Hyaluronic acid in the loose connective tissue is the key component that separates the dense layers and enables gliding between them [45]. A loss of plasma fluid likely leads to an increase in the viscosity of hyaluronic acid and is associated with a reduction in hydrodynamic lubrication and enhanced friction [46]. The HRV2 parameters also showed negative correlations with TLFD but were not significant. Therefore, we hypothesize that the observed reciprocal coupling of HRV1 and TLFD is mainly due to SNS-driven fluid dynamics.

Surprisingly, positive correlations between HRV and TLFD 5 days later were found. With the exception of SD2, which primarily reflects the long-term HRV variations of SNS and PNS [47], only the HRV2 parameters showed significant effects here. It is known from previous studies that PNS activity may reflect the state of recovery after exercise [48, 49]. However, these studies only investigated short-term effects. The finding that there is a positive effect on connective tissue characteristics after 5 days is, therefore, new. Based on the assumption that the underlying mechanism of the negative SNS–TLFD correlation at lag 0 is a loss of fluid in the interstitium, parasympathetic activation with increased plasma volume could be associated with the TLF recovery response as a counter-reaction, as described by the supercompensation theory [50].

Although not significant in the LM, RPE showed a negative correlation with SD2 in the CCF 5 days after higher perceived exertion. Losnegard *et al*. [51], who examined 160 endurance athletes, described a strong relationship between RPE and HRV. However, HRV was measured immediately after exercise, and the relationship with morning resting HRV is probably weaker [52]. Nevertheless, given the results of this study, trainers should be aware that higher levels of RPE can lead to ANS impairment even after longer periods of up to 5 days.

Mood, as measured by the STAI-6, showed no effect on HRV. Dell’Acqua *et al*. [53] demonstrated that depressed mood, rumination, and HRV were interrelated in healthy individuals, with HRV playing a moderating role between the other variables. The association between rumination and depressive symptoms was higher in individuals with reduced SDNN and HF. These results emphasize the complex cascade of interdependencies of biopsychosocial variables. Although no direct cause–effect relationship between mood and HRV was found in this study, it cannot be assumed that there is no moderated or mediated relationship.

There was a significant positive effect of DISE on HRV after 2 days. Two daily stressful events involving family disputes stood out in particular. It is well known from other time series studies in the field of psychoneuroimmunology that the sympathoadrenomedullary system and/or the hypothalamic–pituitary–adrenal axis react dynamically in the days following an emotionally meaningful daily stressor [22–25]. This is accompanied by a delayed decrease in the concentration of neopterin, a cellular immune parameter, about 2 days later [22]. The results of this study are consistent with the observations in the current study, showing that the ANS responds both dynamically and consistently to a significant stressor in the participant. An increase in HRV2, which likely represents the PNS, was also observed, although not significantly. In literature, the SNS/PNS coupling is often described reciprocally [54]. However, SNS/PNS coactivation was observed in the recovery phase after an acute stress task [55], and their coupling, considering time-varying study methods, seems to be rather dynamic and presumably dependent on the type of stressor [56].

Remarkably, HRV1 parameters, particularly LF, was found to mediate the effect of DISE 2 days earlier on the decrease in TLFD by almost 98%. Thus, it could be hypothesized that a negative stressful event, such as a heated argument between the athlete and a family member, leads to a significant decrease in TLFD (DISE_(day 0)_↑ → SNS_(day 2)_↑ → TLFD_(day 2)_↓). It appears that this mechanism was accompanied by an HRV1-coupled HRV2 increase, which could be interpreted as SNS/PNS coactivation. Here, RMSDD, a PNS parameter, mediated the effect of DISE on an increase in TLFD 5 days after the initial LF-mediated reduction in fascial deformability.

There is evidence that the PNS may reduce peripheral proinflammatory cytokines such as interleukin-6, interleukin-1ß and TNF, even if the target organ is not vagally innervated [57]. The PNS is known to regulate proinflammatory cytokines in plasma that correlate with central and peripheral inflammation [58], which strongly influences the viscosity of hyaluronan and its lubricating properties in relation to TLF [59]. Therefore, we hypothesize that the coactivation of the PNS after the daily stressful event is a systemic inflammatory regulatory response leading to a delayed PNS-driven increase in plasma volume [50]. These mechanisms could lead to a kind of supercompensation with increased TLFD, which the mediation analysis probably reflects statistically (DISE_(day 0)_↑ → PNS_(day 2)_↑ → TLFD_(day 5)_↑).

The probably ANS-regulated changes in hydrodynamic tissue lubrication could have implications for the ability of the TLF to support the back muscles. Bojairami and Driscoll [19] found a 75% contribution of the TLF to static spinal stability. Brandl *et al*. [20] found that the TLFD correlates with maximum power in deadlifts in athletes with *r* = 0.88. Considering the results of the current study and adding them to the previous findings on the biomechanical properties of the TLF, the importance of the influence of daily stressful events becomes apparent. It can, therefore, be expected that initially not only a lower performance can be assumed but also a higher risk of injury and/or a higher susceptibility to infection, which has been shown in previous studies [22–25]. Coaches and trainers should be aware of these mechanisms and consider monitoring daily stressful events and HRV during training. Changes in the first few days following such an event along with elevated HRV values could be an indication of fascial tissue restrictions and an increased risk of performance and health impairments.

## Limitations

### The study had a number of limitations

First, there are some studies showing that the SNS variables identified based on cluster analysis, particularly LF, also reflect PNS to some extent and not exclusively sympathetic tone [60, 61]. Therefore, our results could not firmly distinguish between an actual increase in SNS activity or a possible PNS withdrawal. However, the study was able to demonstrate a cause–effect relationship between HRV1 and TLFD. Considering that up to 40% of the total innervation of TLF is sympathetic postganglionic nerve fibers and the lack of description of parasympathetic innervation [9, 11, 62], the authors assume that the immediate effects are SNS-related.

Second, breathing directly influences the measured HRV parameters during data acquisition. Among other things, low respiratory rates below 0.15 Hz lead to an impairment of the LF [63]. In addition, HRV alterations caused by respiratory sinus arrhythmia can occur, a phenomenon that encompasses breathing-related heart rate fluctuations. This can arise in particular when the subject accentuates their own inspiration and expiration [64]. Both were taken into account in this study. The data were analyzed with regard to low breathing frequencies, and the statistics were controlled for breathing rate. In addition, a standardization checklist was used to address all points relating to possible inadequacies in HRV recording [29].

Third, instead of a multichannel electrocardiogram, a consumer chest strap device was used to record HRV data. Data collection during an athlete’s “life as it is lived” required an easy-to-use method. The chest strap sensor was an obvious choice, as the athlete was already familiar with it. Schaffarczyk *et al*. [13] compared the Polar H10 sensor used here with a clinical 12-channel electrocardiogram and found nearly perfect ICCs of 1.0 for heart beats and heart rates at rest and an ICC > 0.85 for the nonlinear short-term scaling exponent alpha 1 of detrended fluctuation analysis, a parameter describing complex cardiac autonomic regulation. These results indicate good concurrency validity of the sensor used in this study with established laboratory devices under resting conditions.

Fourth, the self-reported results are limited to subjective retrospective ratings, which can lead to response bias [65]. Here, future studies should also consider interview-based data collection and hermeneutic interview analysis to identify emotionally meaningful everyday incidents, as seen, for example, in studies in the field of psychoneuroimmunology [22, 66].

Fifth, the observed time series was relatively short (30 days) and limited to one measurement per day. This was mainly due to limitations in the acquisition of HRV data, which is highly dependent on circadian rhythmicity and strict requirements to avoid bias [29]. Therefore, despite using a time series approach, the results of the study only reflect measurements focused on a morning resting state. Previous research suggests that stress-induced ANS changes are more dynamic than the results of this study suggest. One possible option for taking such dynamic processes into account could therefore be the use of long-term (24 hours) HRV recordings [42].

Finally, a particular limitation is the exploratory nature of this study, which is accompanied by design-related boundaries (*n*-of-one). Further replications are, therefore, necessary, and the results may not be generalizable to a larger cohort. However, if the study conditions are close to the natural environment of the participants (“life as it is lived”), the degree of ecological validity of the study increases [22, 66], and thus, the generalizability with regard to the conditions and the protocol [67]. Therefore, the recruitment of a free-living athlete could even be a significant advantage. In contrast to many studies conducted in controlled laboratory settings with RCT-appropriate sample sizes, this study considers the effects of real-world stressors on ANS and TLFD responses in addition to training load, allowing for a more comprehensive view over a specific period of time, such as a mesocycle in this case. In addition, the study provides a comprehensive assessment of stress and HRV markers, providing a holistic approach that enhances understanding of the complex, time-varying interdependencies between daily stressful events, HRV, and tissue characteristics, in contrast to the limited scope of previous studies that focus on data selection at a specific point in time.

## Conclusion

This integrative single-case study of a moderate performance sport climber revealed a direct association between higher HRV1 values, likely indicating SNS activity and lower TLFD. A key finding was the mediating effect of LF, a HRV parameter presumably associated with SNS, on daily stressful events as a predictor of decreasing TLFD. This response was accompanied by a coupled RMSDD increase, which could be considered as SNS/PNS coactivation mediated by a hypothesized supercompensation effect with higher TLFD. The results should encourage coaches and trainers to be aware of these mechanisms and consider monitoring during training to minimize potential additional risk factors for impaired performance and health.

## Supplementary Information

Additional file1 (PDF 502 KB)

## Data Availability

The datasets generated during and/or analyzed during the current study are available from the corresponding author on reasonable request.

## Abbreviations

ANS: Autonomous nervous system
CCF: Cross-correlation function
DISE: Daily inventory of stressful events
HRV: Heart rate variability
LBP: Low back pain
LM: Multiple lagged linear regression model
PNS: Parasympathic nervous system
RPE: Borg rating of perceived exertion
SNS: Sympathetic nervous system
STAI-6: State-trait anxiety inventory six-item short form
TLF: Thoracolumbar fascia
TLFD: Thoracolumbar fascia deformation
