# Supplementary material for: Relations between daily stressful events, exertion, heart rate variability, and thoracolumbar fascia deformability: a case report
Source: J Med Case Rep. 2024 Dec 2;18:589. doi: 10.1186/s13256-024-04935-z (PMC11610301; doi:10.1186/s13256-024-04935-z)
Supplement: Supplementary file 1 — Additional file1 (PDF 502 KB) [file 13256_2024_4935_MOESM1_ESM.pdf]

Supplementary material

Relations between daily stressful events, exertion, heart rate variability and thoracolumbar fascia deformability: An integrative single-case study on a sport climber

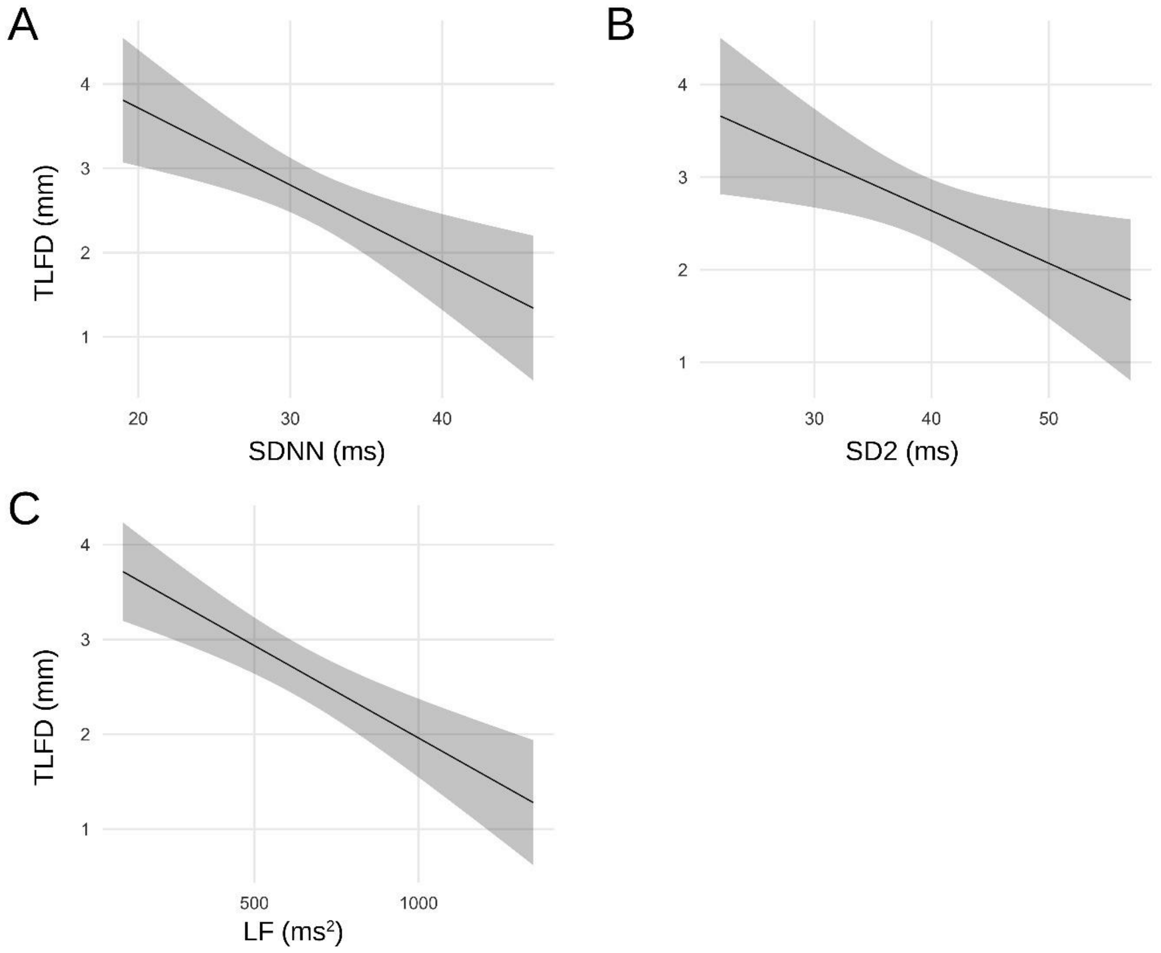

Fig. 1 Linear regression modelling for HRV1 predicting TLFD at time lag 0.
A LM using SDNN as predictor of TLFD. B LM using SD2 as predictor of TLFD. C LM using LF as predictor of TLFD. LM, Linear regression; HRV1, HRV cluster 1; SDNN, Standard Deviation between RR intervals; LF, low frequency band power; SD2, Poincaré parallel standard deviation; TLFD, deformation of the thoracolumbar fascia. The gray shadow shows the 95% confidence interval.

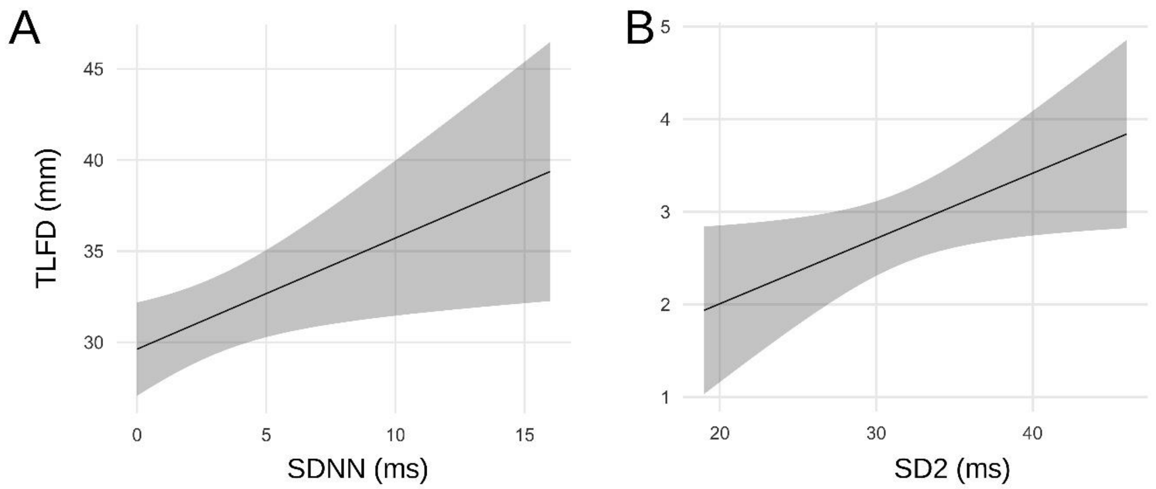

Fig. 2 Linear regression modelling for HRV1 predicting TLFD at time lag 5.
A LM using SDNN as predictor of TLFD. B LM using SD2 as predictor of TLFD. LM, Linear regression; HRV1, HRV cluster 1; SDNN, Standard Deviation between RR intervals; SD2, Poincaré parallel standard deviation; TLFD, deformation of the thoracolumbar fascia. The gray shadow shows the 95% confidence interval.

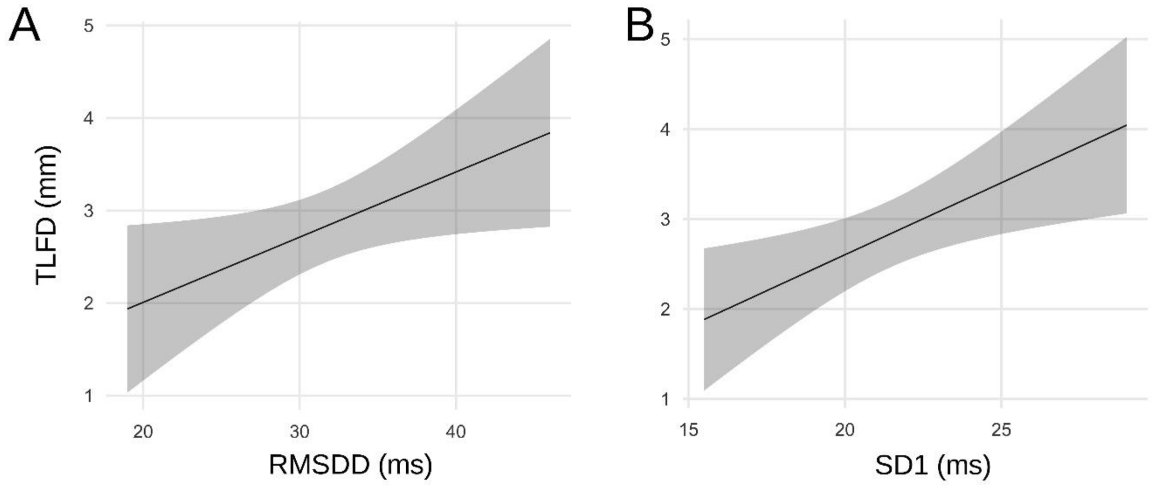

Fig. 3 Linear regression modelling for HRV2 predicting TLFD at time lag 5.
A LM using RMSDD as predictor of TLFD. B LM using SD1 as predictor of TLFD. LM, Linear regression; HRV1, HRV cluster 1; RMSSD, Root Mean Square of successive differences; SD1, Poincaré plot standard deviation perpendicular to the line-of-identity; TLFD, deformation of the thoracolumbar fascia. The gray shadow shows the 95% confidence interval.

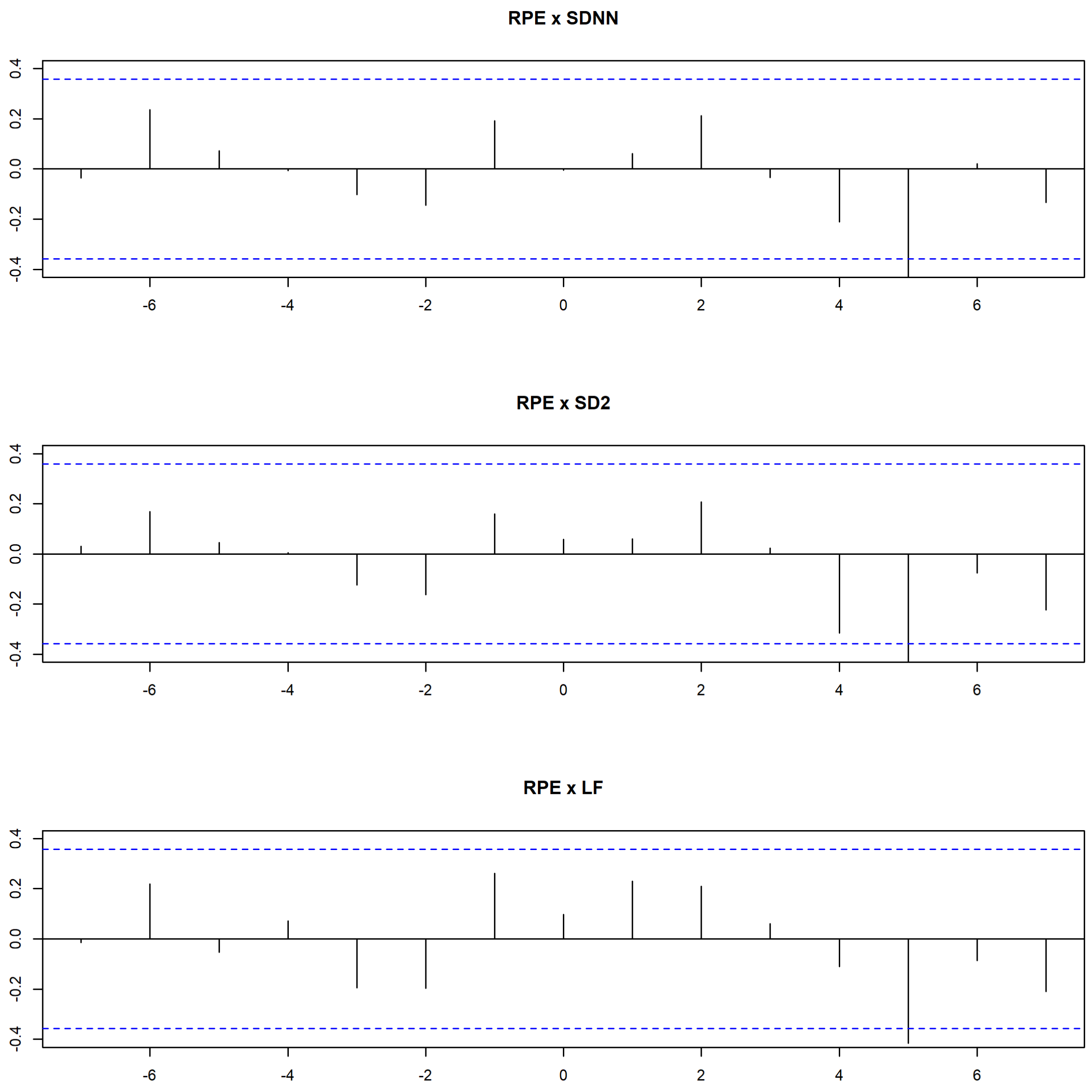

Fig. 4 Cross-correlation function analysis of RPE and HRV1 parameters.
HRV1, HRV cluster 1; RPE, Borg rating scale of perceived exertion; SD2, Poincaré parallel standard deviation; SDNN, Standard Deviation between RR intervals; LF, low frequency band power; SAI, sympathetic activity index.

Table 1 Linear regression modelling for RPE predicting HRV1.

Predictor	Lag	F	R^2	Formula	SE	95% CI Lower	95% CI Upper	t	p^*
SDNN	5	5.64	.34	47.94-1.12x	0.58	-2.32	0.08	-1.94	.207
SD2	5	5.78	.34	61.00-1.53x	0.75	-3.09	0.04	-2.03	.165
LF	5	3.98	.27	1267-66.5x	31.8	-132.5	-0.48	-2.09	.144

HRV1, HRV cluster 1; TLFD, deformation of the thoracolumbar fascia; 95% CI, 95% confidence interval; F, overall model test; SE, standard error; t, t-statistic; SDNN, Standard Deviation between RR intervals; SD2, Poincaré parallel standard deviation; LF, low frequency band power. * Bonferroni adjusted p value.

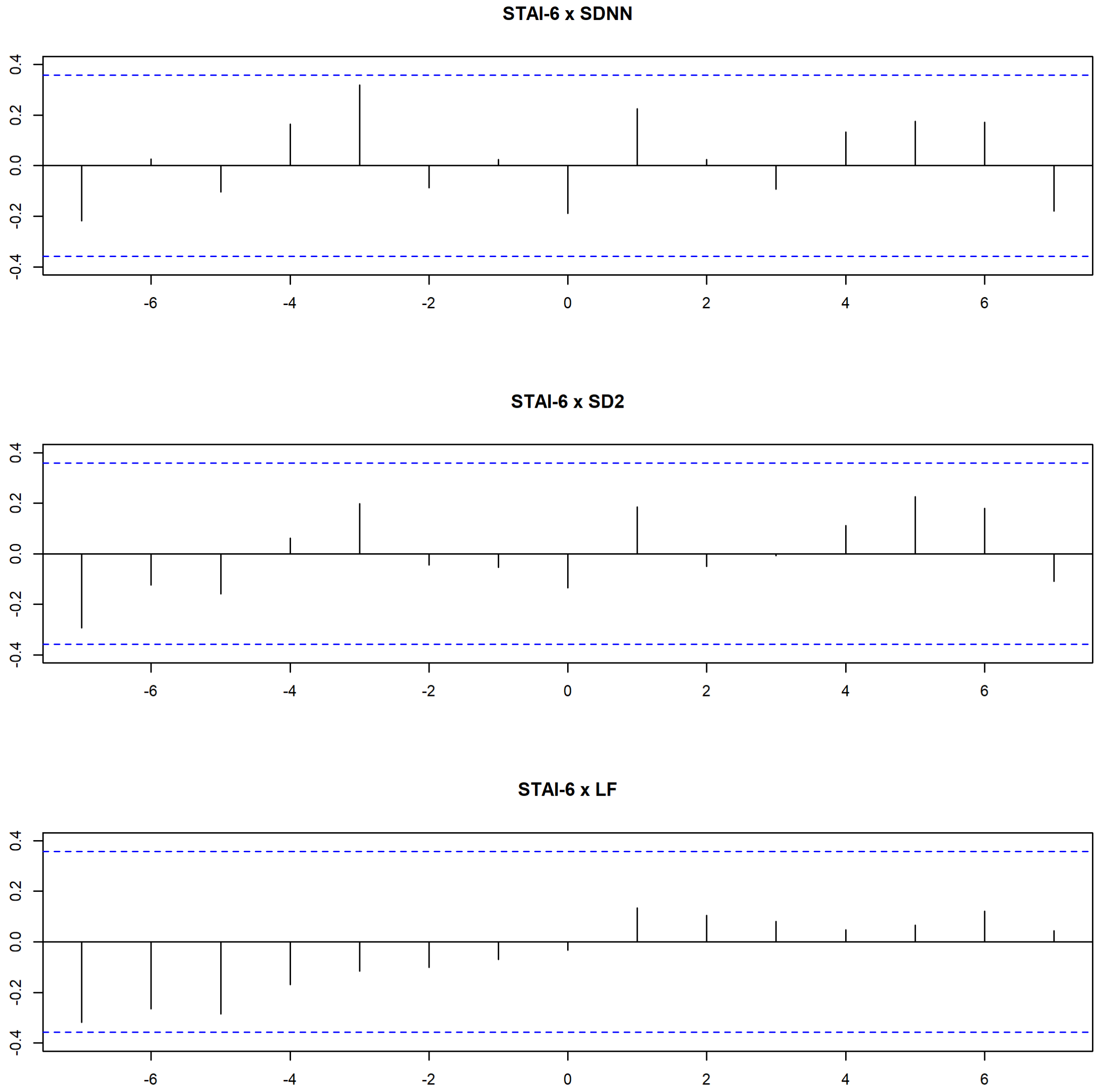

Fig. 5 Cross-correlation function analysis of STAI-6 and HRV1 parameters.
HRV1, HRV cluster 1; STAI-6, State-Trait Anxiety Inventory 6-item short form; SD2, Poincaré parallel standard deviation; SDNN, Standard Deviation between RR intervals; LF, low frequency band power; SAI, sympathetic activity index.

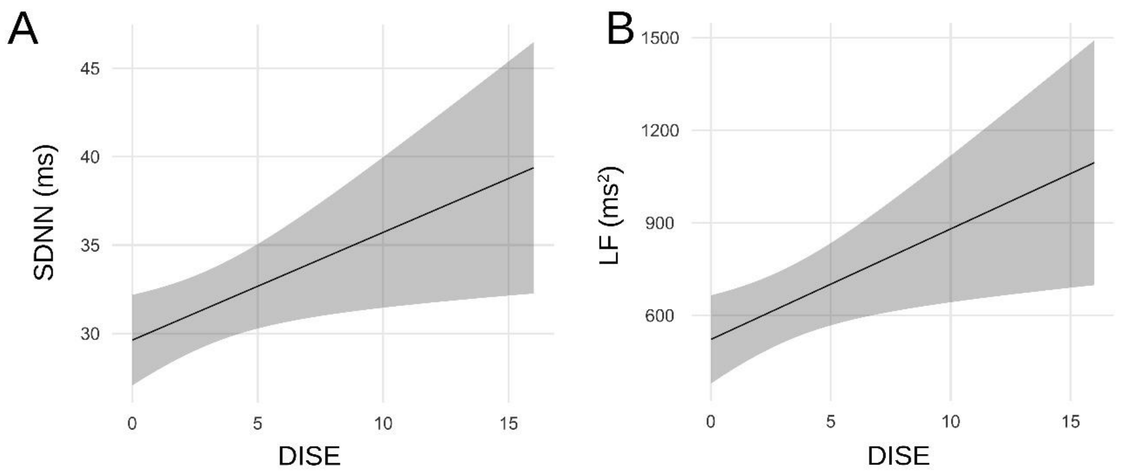

Fig. 6. Linear regression modeling for DISE predicting HRV1 two days later.
A LM using DISE as predictor of SDNN. B LM using DISE as predictor for LF. LM, Linear regression; HRV1, HRV cluster 1; SDNN, Standard Deviation between RR intervals; LF, low frequency band power. The gray shadow shows the 95% confidence interval.

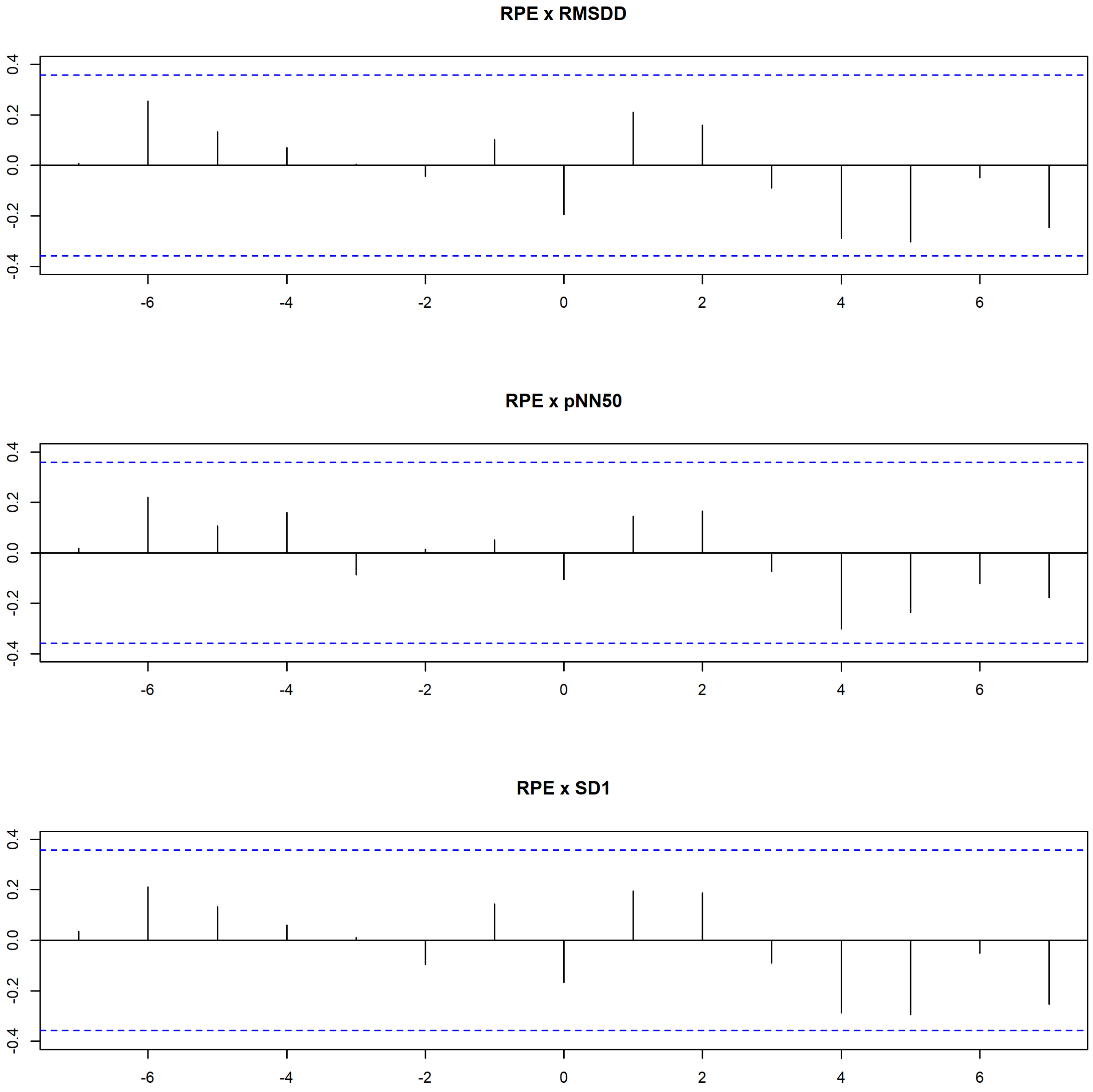

Fig. 7 Cross-correlation function analysis of RPE and HRV2 parameters.
HRV2, HRV cluster 2; RPE, Borg rating scale of perceived exertion; SD1, Poincaré perpendicular standard deviation; pNN50, percentage of successive RR intervals that deviate greater than 50 ms; RMSSD, Root Mean Square of successive differences; PAI, parasympathetic activity index.

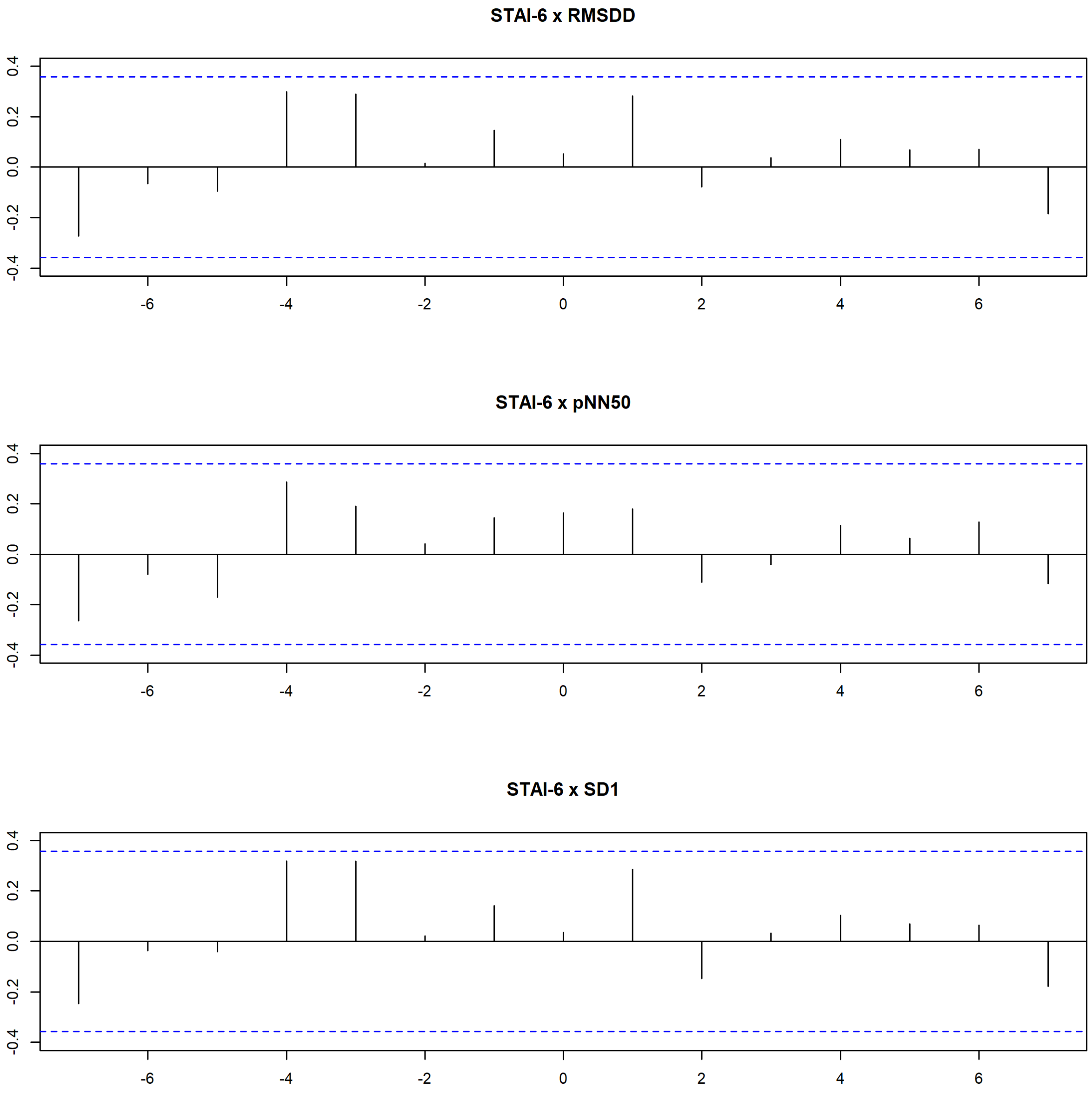

Fig. 8 Cross-correlation function analysis of STAI-6 and HRV2 parameters.
HRV2, HRV cluster 2; STAI-6, State-Trait Anxiety Inventory 6-item short form; SD1, Poincaré perpendicular standard deviation; pNN50, percentage of successive RR intervals that deviate greater than 50 ms; RMSSD, Root Mean Square of successive differences; PAI, parasympathetic activity index.

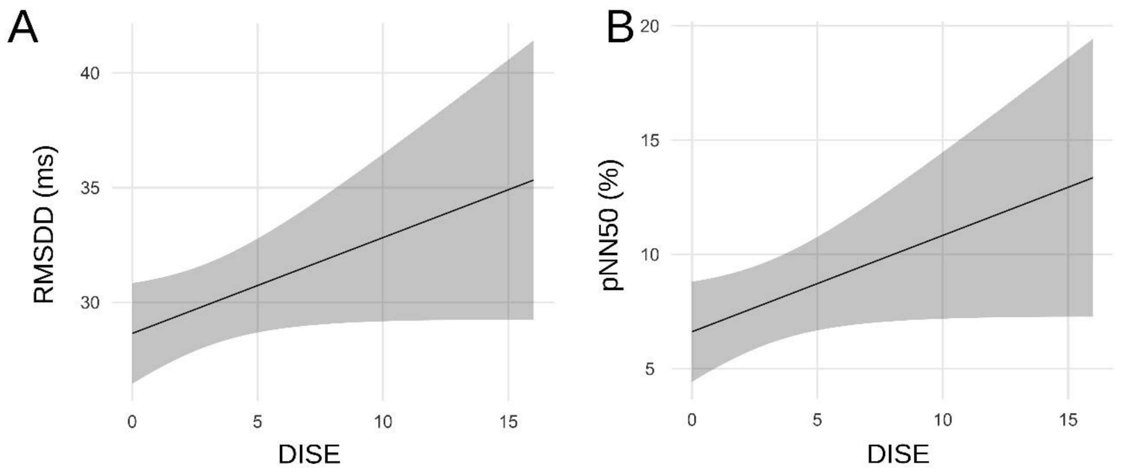

Fig. 9 Linear regression modeling for DISE predicting HRV2 two days later.
A LM using DISE as predictor of RMSDD. B LM using DISE as predictor for pNN50. LM, Linear regression; HRV2, HRV cluster 2; RMSSD, Root Mean Square of successive differences; pNN50, percentage of successive RR intervals that deviate greater than 50 ms. The gray shadow shows the 95% confidence interval.
